# The Liberal Social Values of Swedish Healthcare Providers in Women’s Healthcare: Implications for Clinical Encounters in a Diversified Sexual and Reproductive Healthcare

**DOI:** 10.3389/ijph.2022.1605000

**Published:** 2022-07-11

**Authors:** Lise Eriksson, Andrey Tibajev, Irina Vartanova, Pontus Strimling, Birgitta Essén

**Affiliations:** ^1^ Department of Women’s and Children’s Health, Faculty of Medicine, Uppsala University, Uppsala, Sweden; ^2^ Institute for Futures Studies, Stockholm, Sweden

**Keywords:** migration, gender equality, migrant healthcare, sexual and reproductive healthcare, sexual and reproductive rights, values

## Abstract

**Objectives:** Women’s healthcare is a potential source of cross-cultural conflicts. Diverging values between healthcare providers and patients challenges the provision of culturally sensitive care and meeting migrant women’s needs. The aim is to investigate healthcare providers’ values in relation to sexual and reproductive rights, gender equality, migration and religion in Swedish sexual and reproductive healthcare.

**Methods:** A national cross-sectional study was carried out. The questionnaire was distributed through a non-probability sample to midwives or other nurses, gynaecologists and obstetricians, and hospital social workers (*n* = 1,041). Through descriptive statistics, we mapped their values, comparing healthcare provider data to external representative population survey data.

**Results:** Healthcare providers in sexual and reproductive healthcare displayed homogeneous liberal social values, being permissive towards sexual and reproductive rights and restrictive against gender-based violence. They were for gender equality, expressed low anti-immigrant sentiments, and had even more liberal values than the Swedish population and a demographically comparative sub-population.

**Conclusion:** Individuals with very liberal values are selected to work in Swedish sexual and reproductive healthcare. Healthcare providers need self-reflexivity to avoid conflicts in clinical encounters in a diversified society.

## Introduction

The human rights perspective of sexual and reproductive rights includes reproductive decision-making free of discrimination, coercion and violence [1]. The Guttmacher-Lancet Commission emphasized; “All individuals have a right to make decisions governing their bodies and to access services that support that right” [2]. Hence, sexual and reproductive healthcare services, including abortion and contraceptive counselling, are potential contentious spaces and sources of cross-cultural conflicts and conflicts between private and professional values. In the context of migrant healthcare, diverging values between healthcare providers (HCPs) and patients challenges the provision of culturally sensitive care and meeting migrant women’s specific health needs.

Healthcare providers relate to the politicized area of sexual and reproductive rights, gender equality, migration and religion with influence by personal values, ideologies, professional ethos, laws and policy guidelines. By “professional ethos” we refer to a set of internalized professional norms and values guiding professional practice. International studies have shown that moral and religious values may affect HCPs’ clinical decisions and patient care [3, 4]. By international comparison, people in Sweden display the most liberal and individualistic values on reproduction and sexuality [5]. However, values of Swedish HCPs are under-researched, despite contributing to tensions in healthcare between equality and cultural diversity, and between secularity and religious diversity. Previous studies have shown that the dilemma of gender equality and cultural diversity in Swedish healthcare encounters involves tensions between the values of cultural and religious diversity and liberal, individualistic values on sexual and reproductive rights matters [6, 7]. By investigating values at the intersection of the individual, professional and policy levels, this study also contributes to research on structural asymmetries in healthcare access in Sweden and other European countries [8]. For example, qualitative studies have found indications of ethnic minority healthcare staff and healthcare users experiencing racism in Swedish healthcare [8]. In multicultural consultations in women’s healthcare, HCPs’ awareness of their own values and patients’ values is of great importance for successful consultations by preventing misunderstandings and thus suboptimal care [6, 9, 10].

Sweden became a country of net immigration in the post-war years and after an acceleration of immigration in the 21st century, a fifth of the Swedish population are currently foreign born with the Nordic countries, former Yugoslavia, Syria, and Iraq being the most common backgrounds [11]. Healthcare providers caring for Sweden’s diverse population use policy strategies to handle differences in language, health literacy, cultural norms and values [6]. Migrants’ public health research suggests that multicultural integration policies, characterising Sweden, have a positive effect on self-rated health of migrants in comparison to assimilationist and exclusionist policy models [12]. In light of Sweden’s changed demography due to immigration from low-income countries, Swedish policies emphasize, on the one hand, that public welfare institutions should promote cultural and religious diversity [13]. Sweden’s attempts to address health inequalities among migrants include promotion of culturally sensitive care, which takes into account how perceptions of sexual and reproductive rights and gender equality are influenced by cultural norms [14]. The purpose of culturally sensitive care is to improve communication between HCPs and diverse populations, enhance migrants’ health literacy, and acknowledge the impact of the migration process on health [14]. On the other hand, as part of the Swedish Government’s gender mainstreaming program, HCPs are encouraged to incorporate gender equality perspectives [15].

Sexual and reproductive healthcare is internationally the foremost medical field allowing conscientious objection [16]. Unlike law in most EU countries, Swedish policy does not grant HCPs conscientious objection and protection of women’s reproductive rights has broad societal support. Since the 1990s, access to abortion care is better than in most Western countries, because midwives can provide counselling and medical abortion—although prescribed by physicians—and women do not need to travel for abortions [3]. Historically, controversies surrounded abortion in Sweden since the introduction of abortion for socio-medical reasons in 1946 until the 1975 Abortion Act permitting abortion on demand [17, 18]. During these decades, some prominent gynaecologists joined Christian conservatives in campaigns against abortion [18].

Most professional associations of HCPs were open to liberalization of abortion law in early 1970s [17]. In statements to the government report The right to abortion (1971) the Swedish Medical Association, the Swedish Gynecological Association and the Swedish Hospital Social Workers’ Association advocated women’s decision-making power in early pregnancy. However, the Swedish Association of Midwives was critical of women’s statutory right to abortion and advocated established indications for abortion [17, 19]. Since the 1980s, there has been strong support for abortion on demand, and Swedish anti-abortion campaigns demanding a conscience clause in the 1990s had no political success [20]. Still today, heated political debates on abortion and freedom of conscience occur in several European countries in relation to HCPs’ right to refuse to provide or refer for abortion [21]. To prevent conflicts and discrimination, we need more knowledge about HCPs’ values about sexual and reproductive rights, gender equality, migration and religion in relation to policies governing sexual and reproductive healthcare.

The aim is to investigate self-expressed values in relation to sexual and reproductive rights, gender equality, migration and religion among Swedish HCPs in sexual and reproductive healthcare in comparison with the Swedish population. We analyse the following research questions: 1) What are the values in relation to sexual and reproductive rights, gender equality, migration, and religion among HCPs in sexual and reproductive healthcare? 2) To what extent do these values among HCPs differ from values among the Swedish population in general, and if so, why do they differ? We expect that the differences between the values among HCPs and the Swedish population correlate with socio-demographic attributes, selection into healthcare professions, as well as professional ethos and clinical experience.

## Methods

### Study Setting

The target group was midwives or other nurses, gynaecologists and obstetricians, and hospital social workers within sexual and reproductive healthcare in Sweden. This includes outpatient and inpatient care in gynaecology, obstetric care and reproductive medicine. Most nurses are midwives, and most physicians in sexual and reproductive healthcare are gynaecologists and obstetricians. Social workers in Swedish healthcare are since 2019 licensed as healthcare counsellors. We refer to both licensed and unlicensed social workers in healthcare by the term “hospital social workers.” When we onwards use the term HCPs, we refer to Swedish HCPs in sexual and reproductive healthcare.

### Study Design

We conducted a cross-sectional study using an online questionnaire. The questionnaire was pilot tested twice—first with 20 respondents within the target group including follow-up qualitative interviews, second with 200 respondents outside the target group to test the used measures. All respondents were anonymous, and no identifying data were collected [22].

### Variables

The questionnaire included questions about HCPs’ demographic and work characteristics, as well as values within sexual and reproductive rights, gender equality, migration and religion. To allow for comparison with the general Swedish population, value questions were taken from the European Social Survey (ESS2002), European Values Study (EVS2017), International Social Survey Programme (ISSP2018) and World Values Survey (WVS2011) where applicable [23–26]. Table 1 displays an overview of the included value questions with information about data source for the Swedish comparison group. As noted, some of the questions did not appear in the Swedish population-wide surveys.

**TABLE 1:** Overview of variables, survey questions about values, and data sources for the Swedish comparison group. MigraMed Healthcare Providers Study, Sweden, 2021.

Variable	Question	Source	Swedish data
Justifiable: IVF	Assisted reproduction or *in vitro* fertilization (IVF)	WVS^a^	
Justify: International adoption	To become a parent through adoption *via* Adoptionscentrum from a country where it is legal	Own	
Justify: Commercial surrogacy	To become a parent through commercial surrogacy in a country where it is legal	Own	
Justifiable: Abortion	Abortion	EVS	EVS2017
Justifiable: Homosexuality	Homosexuality	EVS	EVS2017
Justifiable: Sex before marriage	Having casual sex before marriage	WVS	WVS2011
Justifiable: Underage teenage sex	Underage teenagers having casual sex	Own	
Justifiable: Divorce	Divorce	EVS	EVS2017
Justifiable: Spank children^b^	Parents smacking their children	WVS	WVS2011
Justifiable: Beat wife^b^	For a man to beat his wife	WVS	WVS2011
Justifiable: Prostitution (selling)	Prostitution, to sell one’s body for money	Own	
University for boys^c^	University is more important for a boy than for a girl	EVS	EVS2017
Jobs for men^c^	Jobs scarce: Men should have more right to a job than women	EVS	EVS2017
Men better political leaders^c^	Men make better political leaders than women do	EVS	EVS2017
Migrants: Live here	Allow many/few immigrants of different race/ethnic group from majority	ESS	ESS2002
Migrants: Enrich culture	Country’s cultural life undermined or enriched by immigrants	ESS	ESS2002
Migrants: Better country	Immigrants make country worse or better place to live	ESS	ESS2002
Religiosity^b^	Would you describe yourself as... [extremely religious—extremely non-religious]	ISSP	ISSP2018
Religious affiliation	Do you belong to a church or another religious denomination? Which?	ISSP	ISSP2018

a Question in standard World Values Survey questionnaire, however not in the European version (EVS2017) that includes data for Sweden.

b Reversed (both datasets) in analyses.

c Reversed (HCP data) in analyses.

Note: Data description and codebook for the HCP survey available [22].

We define the term liberal social values as being permissive within matters of sexual and reproductive rights, against gender-based violence, for gender equality, and for immigration. Some social value items are therefore reversed in the following analyses (see Table 1) so that all higher values imply more liberal. The questions had different scales. If not stated otherwise, all value items are scaled to 0–1 to facilitate comparison.

### Data Collection

Data was collected between January and May 2021 with a non-probability sample by distributing the questionnaire through the target population’s workplaces and through professional associations and interest groups for Swedish midwives and gynaecologists. At the data collection stage, all participants were welcome to answer the questionnaire without any exclusion based on workplace or profession. This resulted in a total sample of 1,257 respondents. The analytical sample is constructed by excluding respondents who do not fit the target population: either working in other fields of medicine than sexual and reproductive healthcare, in another profession, or being of retirement age (above 67). This produces an analytical sample of 1,041 respondents, covering 594 midwives, 411 physicians and 36 hospital social workers (Supplementary Table S1). Based on official statistics [27], and using the number of midwives working in healthcare and gynaecologists/obstetricians under the age of 65 as baselines, the analytical sample covers roughly nine and 30%, respectively, of the target populations. The difference was likely due to more effective marketing by the professional association for gynaecologists, distributing the survey to all members by e-mail, and a higher proportion of Swedish gynaecologists being members in comparison to the association for midwives. To our knowledge, there are no statistics of hospital social workers in sexual and reproductive healthcare. Tests of the values for the each of three professions in the sample revealed no substantial differences between them, and we therefore refrain from dividing up the presentation of results by profession.

### Statistical Analysis

The research questions are analysed using descriptive statistics, comparing means of values and distributions between HCPs and the Swedish population. Almost all respondents in the HCP sample are women and, by definition, highly educated with at least a bachelor’s degree (including midwives), and no older than 67 years old (Supplementary Table S1). Demographic variables such as gender, education, and age are known to correlate with social and religious values [28–30]. To study whether the values of HCPs are different from what can be expected given their demographic composition, i.e., to answer the second research question, a representative sub-sample of the Swedish population that only includes highly educated (bachelor and above) women under the age of 67 is created (Supplementary Table S2, for demographic variables of the Swedish population). If HCPs and the sub-sample exhibit the same value patterns, then the values of HCPs are explained by their demographic composition. However, if differences in values are found between the two groups, then the explanation for HCPs’ values must be found in relation to their profession. Additional analyses are performed for issues on which HCPs stand out in comparison to the sub-sample to study whether their values vary with clinical years. We do not assume any specific correlation pattern and consequently use locally weighted scatterplot smoothing (LOWESS) for variables we treat as continuous (all but religious affiliation) and a multinomial logistic regression with clinical years and clinical years squared for the nominal variable religious affiliation. LOWESS is a non-parametric analysis presenting the data as it is, and multinomial logistic regression does not assume any specific relation between the included categories.

### Ethical Approval

Ethical approval was gained by the Regional Ethical Committee (Dnr 2018/425) and by the Swedish Ethical Review Authority (Dnr 2020-07187). Questions were designed to minimize the risk of discomfort and invasion of privacy, and informed written consent was obtained.

## Results

### HCPs’ Self-Expressed Values

Starting with the first research question on HCPs values, the respondents displayed homogeneous values, often at the extremes of included scales (Supplementary Table S1). Regarding questions whether sexual and reproductive rights matters are never or always justifiable, HCPs found on average that IVF (average of 0.8 on a 0–1 scale), adoption (0.8), abortion (0.9), homosexuality (1.0), sex before marriage (0.9), underage teenage sex (0.8) and divorce (0.9) were all almost always justifiable. Likewise, parents spanking their children and gender-based violence were universally considered never justifiable with averages of 1.0 on both items on a reversed scale. More mixed results were recorded for commercial surrogacy (0.4) and prostitution (0.2).

There was even less variation in answers relating to gender equality values. HCPs strongly disagreed to statements that one should prioritize boys’ university education (1.0) and men’s right to work (1.0), and that men are better political leaders than women (1.0). HCPs also expressed low anti-immigrant sentiments, wanting to allow more migrants of different race/ethnicity to live in Sweden (0.9), and thinking that migrants enrich Swedish culture (0.8) and make the country a better place to live in (0.7).

Religion was measured in two ways. First, a self-estimation of religiosity, originally on a scale with seven steps, ranging from extremely non-religious to extremely religious, and second, regarding membership in a church or religious denomination. On average, HCPs defined themselves as neither religious nor non-religious (0.4). About 71% were not members of any denomination. In addition to non-affiliation, membership in the Church of Sweden was most common, while other Christian and other denominations were quite rare.

### Comparison Between HCPs and the Swedish Population

**FIGURE 1:**
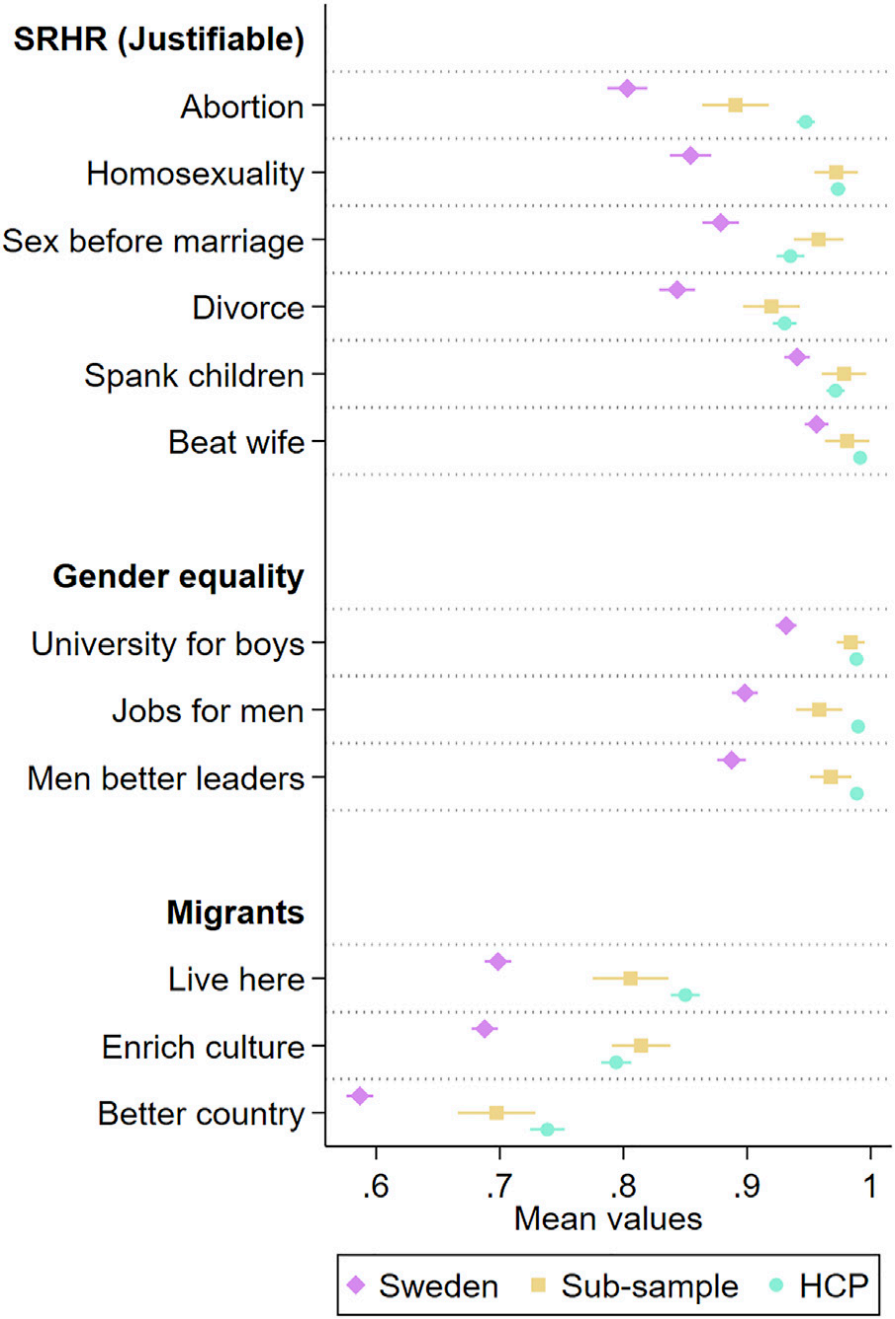
Values in relation to sexual and reproductive rights, gender equality and migration for the Swedish population, a sub-sample of highly educated women no older than 67, and healthcare providers in sexual and reproductive healthcare. Average values with 95 percent confidence intervals. MigraMed Healthcare Providers Study, Sweden, 2021.

The second research question concerns HCPs values in relation to the Swedish population. As previously noted, HCPs have a particular demographic composition that might be correlated with their values, therefore, we created the representative Swedish sub-sample of highly educated women no older than 67 as a comparison. Consistent with previous research on correlation between demographic characteristics with social values [28–30], there are clear differences between average Swedish values and the values of the sub-sample regarding sexual and reproductive rights, gender equality and migration (Figure 1). T-tests reveal that the difference in average values is statistically significant (*p* < 0.001, Supplementary Table S3) for everything but gender-based violence and spanking children, with the sub-sample being more permissive of issues in sexual and reproductive rights, more for gender equality, and less anti-immigrant.

Comparing the sub-sample and HCPs across these issues, only abortion stands out where HCPs have different values than expected given their demographic composition. On the question whether abortion is justifiable, HCPs have a higher value. For all other issues, there are no real differences.

**FIGURE 2:**
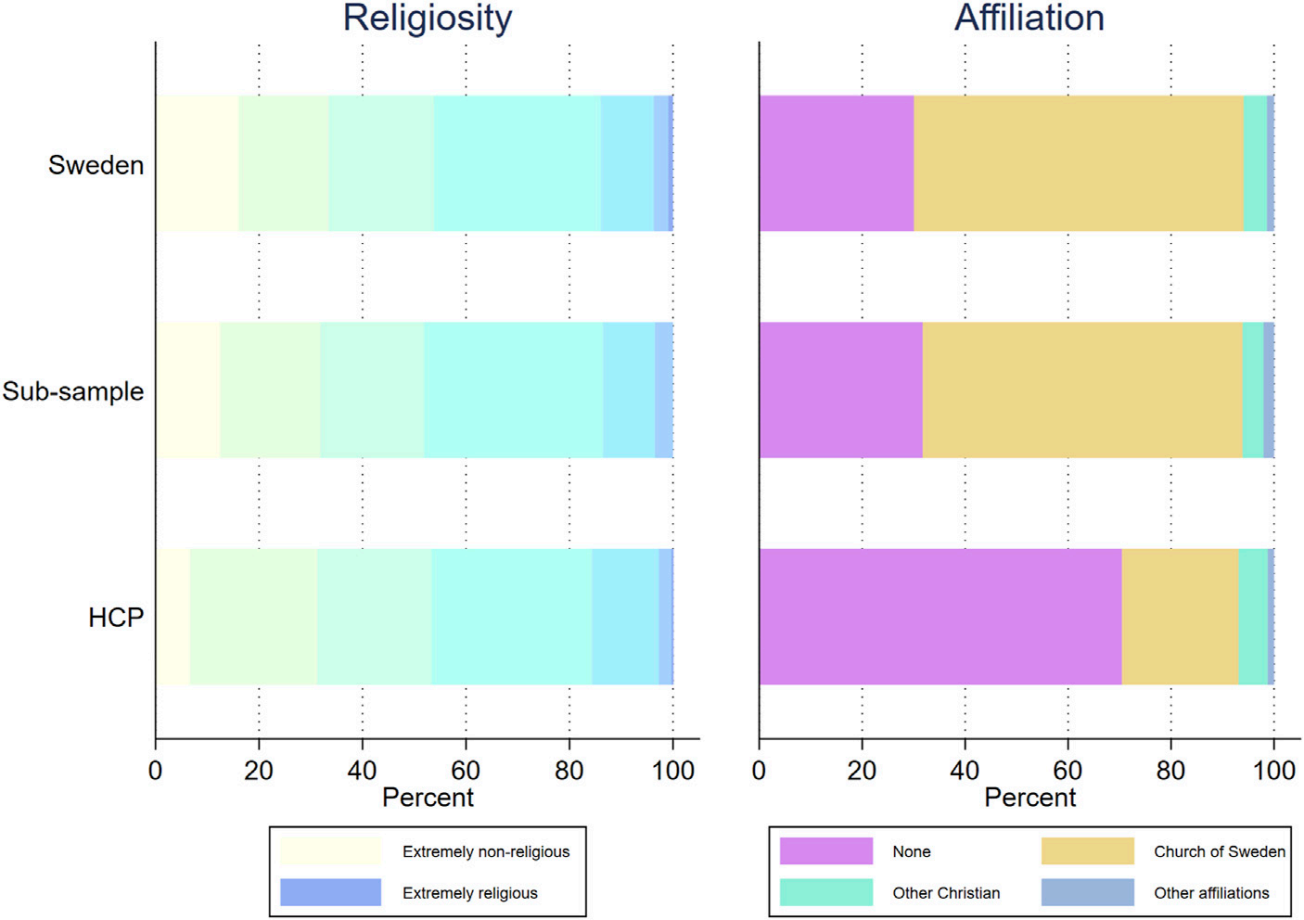
Religiosity and religious affiliation for the Swedish population, a sub-sample of highly educated women no older than 67, and healthcare providers in sexual and reproductive healthcare. MigraMed Healthcare Providers Study, Sweden, 2021.

In contrast to the measures of social values, there is no difference in either membership or religiosity between the general Swedish population and the sub-sample (Figure 2; Supplementary Table S3). Seventy percent of the Swedish population and 68% of the sub-sample are members in a religious denomination and both samples have an average religiosity of 3.2.

Comparing all three samples, HCPs have a very similar distribution as the other two for religiosity. However, as an unexpected result, they are in stark contrast regarding religious membership, particularly HCPs’ lower degree of membership in Church of Sweden.

### The Relationship Between Profession and Values

The results indicate that HCPs have largely the expected values given their demographic composition, with two exceptions. First, they find abortion even more justifiable than the already high value among the sub-sample, and second, they are largely less members in a religious denomination. These differences can be caused by both a causal mechanism, i.e., having a profession and being at a workplace affects values, and selection, i.e., specific individuals choosing or being selected for these professions and workplaces based on personal values they already had.

We test these causes by analysing whether there is variation in abortion values and religious affiliation across clinical years. While it is not possible to make a direct test of causality with our cross-sectional sample, clinical years can function as an indirect test of change over time. That is, if there is a causal effect of profession and workplace, there should be a correlation between, on the one hand, clinical years and, on the other, values and affiliation, with more senior HCPs finding abortion to be more justifiable and the least have a religious affiliation.

**FIGURE 3:**
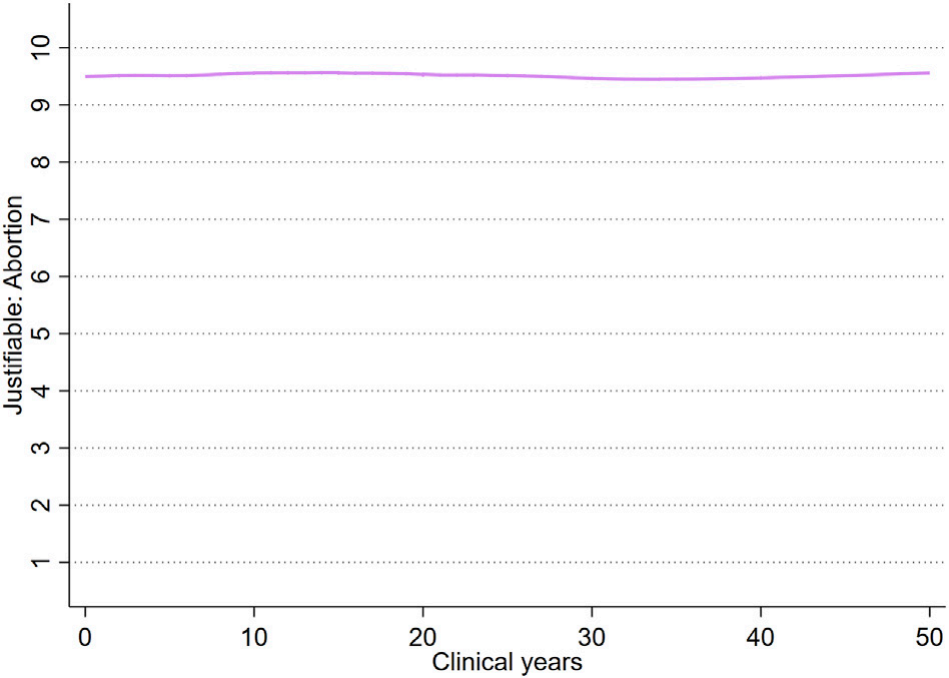
Locally weighted scatterplot smoothing (LOWESS) of Justifiable: abortion values over clinical years. MigraMed Healthcare Providers Study, Sweden, 2021.

**FIGURE 4:**
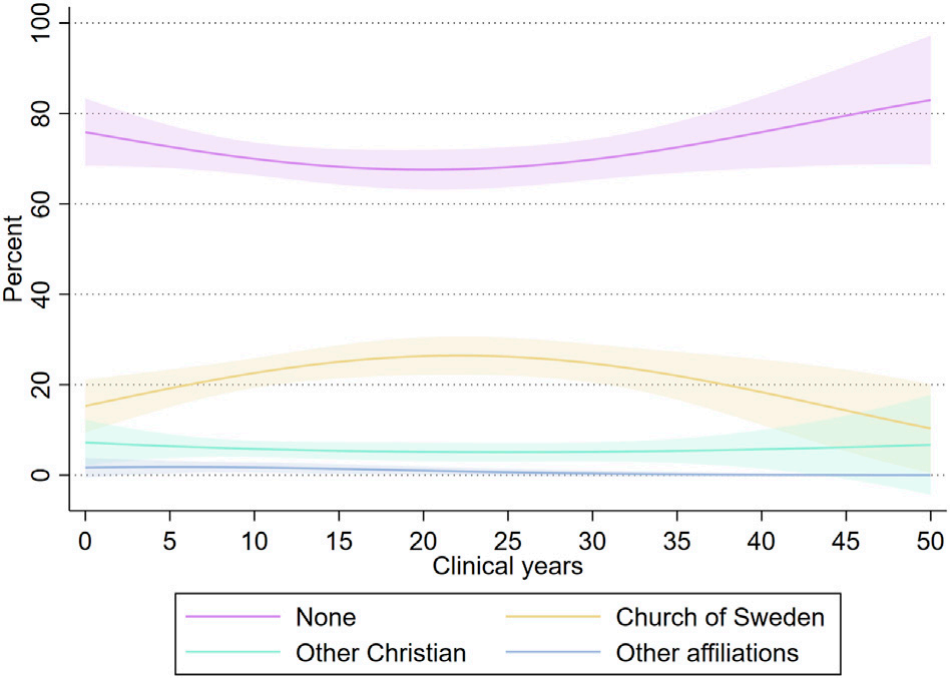
Predicted probability with 95 percent confidence intervals from robust standard errors of religious affiliation over clinical years. MigraMed Healthcare Providers Study, Sweden, 2021.

For abortion values, Figure 3 contains the results from a LOWESS. The result is that no such correlation with time exists, and respondents found on average abortion to be equally justifiable regardless of years in the profession. For religious affiliation, Figure 4 displays predicted probabilities of each affiliation over clinical years after a multinomial logistic regression with clinical years and clinical years squared as independent variables (results in Supplementary Table S4). Results indicate some variation over clinical years, but nothing that supports a negative relationship between affiliation and clinical years. In both cases, the indication is that individuals with specific values of abortion and religious choices are different from their demographic counterparts already at the start of their professional careers, i.e., are selected into these professions.

## Discussion

This is the first large-scale values survey conducted in Swedish sexual and reproductive healthcare. The study highlights complex relationships between individual and professional values. In theory, HCPs as individuals may choose to follow the hospital’s policy despite subjective values contrary to healthcare’s norm system. Our major findings demonstrate high homogeneity in liberal values among HCPs. Thus, there is a strong correlation between HCPs’ personal values and Swedish healthcare’s policies on gender equality and equal care. We conclude that HCPs in sexual and reproductive healthcare share a professional ethos of written and unwritten rules prioritizing gender equality and liberal values in relation to sexual and reproductive rights, religion and migration, i.e., they did not hold anti-immigrant views.

Longitudinal results of WVS and EVS suggest that “individual choice norms,” such as acceptance of gender equality, divorce, abortion and homosexuality, change faster than other values [5]. The change from traditional “pro-fertility norms,” emphasizing traditional gender roles and linking sexual behaviour to reproduction, towards individual choice norms is fastest in high-income societies [5]. Sweden is a forerunner in this trend by having the most liberal and individualistic sexual and reproductive rights and gender equality values in international comparison [5].

As an extended analysis of abortion values and religious affiliation, the comparison group was changed based on occupation instead of demography, comprising HCPs in outpatient and inpatient care in all fields of medicine and coded based on occupational codes (ISCO) in the two relevant data sources. In accordance with the main analysis, HCPs working within sexual and reproductive healthcare stand out in their permissiveness towards abortion and religious non-affiliation, implying a specific connection to the chosen workplace and not only the medical professions (Supplementary Tables S5, S6).

The difference in abortion values between the sub-sample and HCPs is intuitive. In general, HCPs in sexual and reproductive healthcare are expected to have the same position to, e.g., homosexuality, divorce or migrants as the demographically similar sub-sample. However, they have a different relationship to abortions based on their profession and workplace. Previous research has found that HCPs and medical students objecting to abortion or contraceptive counselling are not employed in Swedish sexual and reproductive healthcare due to the policy ban on conscientious objection, but they can usually find work in another field of medicine [3]. In addition to self-selection whether to work in sexual and reproductive healthcare, this may explain why HCP’s abortion values are more liberal compared to the Swedish population and the sub-sample. Another explanation is that clinical work may increase abortion permissiveness in Swedish sexual and reproductive healthcare with its strong gender equality policy [6, 7]. However, our findings demonstrated no variation in abortion values in relation to clinical years. For future research, longitudinal results are required to determine which explanation is correct.

Of the survey questions that we constructed, the distribution of answers about adoption and underage teenage sex follow the liberal pattern for other values, while results for questions about surrogacy and prostitution (selling sex) differ. Attitudes to surrogacy and prostitution are paradoxical in the Swedish context. Despite cross-border surrogacy being increasingly common among Swedes, arguments about women’s reproductive and bodily autonomy are used in public debates to criticise surrogacy as exploitation, which also is one of the arguments against selling sex [31, 32]. HCPs in sexual and reproductive healthcare had very permissive attitudes towards IVF, but in line with previous research, they had mixed attitudes towards surrogacy, which has never been practiced in Swedish healthcare [33, 34]. A previous survey found that 63% of physicians in Swedish sexual and reproductive healthcare were positive or neutral towards permitting altruistic surrogacy in healthcare, but 60% agreed that surrogacy involves exploitation of women’s bodies [33]. Prostitution is difficult to define with our definition of liberal social values, because both higher and lower values can be liberal depending on arguments used for each position. Sweden was a pioneer in 1999 through the enactment of a law criminalizing sex purchase [35]. Previous research suggests that criminalization of sex purchase has broad societal support, while attitudes to selling sex are mixed in Sweden [35, 36].

Religiosity has during recent decades declined in most high-income countries [5]. Sweden’s population is religiously diverse due to immigration. However, membership in the Church of Sweden remains high despite Sweden’s low levels of religiosity in terms of beliefs [37, 38]. Our comparative results show low levels of religiosity among HCPs within sexual and reproductive healthcare as well as in the Swedish population, but a significantly smaller proportion of the HCPs are members of Church of Sweden. This low propensity to be religiously affiliated did not vary much across clinical years, which again indicates a selection mechanism at work.

Previous research suggests that person-centeredness correlates well with multiculturalist ideologies emphasizing religious and cultural diversity, but conflicts may emerge between promotion of cultural sensitivity and gender equality [6]. Hence, in sexual and reproductive healthcare, HCPs’ homogeneity in liberal values and their incorporation of gender equality perspectives may contribute to tensions in healthcare encounters with migrants from countries with different values. Reflexivity can help HCPs to observe and question taken-for-granted assumptions in clinical encounters [6, 39]. Reflecting on one’s own values is the first step in counteracting conflicts in healthcare encounters. To ensure equal care, HCPs need self-reflexivity about their own values when encountering migrants, and their presumed more restrictive values in relation to sexual and reproductive rights and equality. They also need to reflect on where the limit goes between accommodating differences and providing equal treatment. This is especially important given that HCPs have very liberal values in comparison to their patients and society. Our results pointing towards a selection mechanism based on values and religious affiliation indicate that recruiting healthcare students with religious conservative ideas may entail a future challenge. Further studies are necessary to investigate value conflicts in healthcare encounters and the impact of migration on HCPs’ values.

### Strengths and Limitations

The benefits of the study were a large sample size (*n* = 1,041) and consistent results in comparisons of HCPs in sexual and reproductive healthcare, the general Swedish population and the sub-sample. Due to the non-probability sample and lack of longitudinal data, our study focuses on descriptive results and makes few causality claims. However, the analyses of abortion values and religious affiliation all point to a selection into the specific combination of professions and workplace as the main mechanism for the difference HCPs and their demographic peers. Our sample has different coverage rates between the included professions and an underrepresentation of HCPs who had immigrated; 12% in the sample compared to approximately 23% in Sweden [40]. In values surveys, there is also a risk of social desirability bias. The underrepresentation of immigrants and possible bias risks the answerers of being too liberal. We have not explicitly tested for the magnitude of this possibility. However, the fact that there was strong alignment in values between the respondents and the Swedish sub-sample implies that there is no great bias present in the data.

### Conclusion

Our findings demonstrate that HCPs in Swedish sexual and reproductive healthcare share a professional ethos emphasizing gender equality, and are homogeneous in their secular liberal values in relation to sexual and reproductive rights, gender equality, migration and religion. They did not hold anti-immigrant views and had zero tolerance to gender-based violence and child spanking. Compared to the Swedish population, HCPs displayed even more liberal social values. Their values correspond to Swedish women of similar demographic background, with the exception of having more liberal abortion values and being less religiously affiliated. The results point towards a selection mechanism of individuals with very liberal values choosing or being selected to work in Swedish sexual and reproductive healthcare. Hence, HCPs need self-reflexivity to provide culturally sensitive care and to avoid conflicts in clinical encounters in a diversified society.
