# Supplementary material for: The Liberal Social Values of Swedish Healthcare Providers in Women’s Healthcare: Implications for Clinical Encounters in a Diversified Sexual and Reproductive Healthcare
Source: Int J Public Health. 2022 Jul 11;67:1605000. doi: 10.3389/ijph.2022.1605000 (PMC9309245; doi:10.3389/ijph.2022.1605000)
Supplement: Supplementary file 1 [file DataSheet1.docx]

**Supplementary material for ‘The Liberal Social Values of Swedish Healthcare Providers in Women’s Healthcare: Implications for Clinical Encounters in a Diversified Sexual and Reproductive Healthcare’. International Journal of Public Health**

**TABLE S1.** Descriptive statistics for the healthcare providers survey. MigraMed Healthcare Providers Study, Sweden, 2021.

| **Variable** | **N** | **Range** | **Mean** | **Sd** |
| --- | --- | --- | --- | --- |
| Education |  |  |  |  |
| Midwife/nurse | 1041 | 0/1 | 0.571 |  |
| Physician | 1041 | 0/1 | 0.395 |  |
| Hospital social worker | 1041 | 0/1 | 0.035 |  |
| Gender |  |  |  |  |
| Male | 1035 | 0/1 | 0.066 |  |
| Female | 1035 | 0/1 | 0.934 |  |
| Clinical years | 1024 | 0–50 | 17.539 | 11.319 |
| Age | 1041 | 24–67 | 46.252 | 10.860 |
| Justify: IVF | 947 | 0.22–1 | 0.823 | 0.158 |
| Justify: International adoption | 951 | 0–1 | 0.837 | 0.191 |
| Justify: Commercial surrogacy | 941 | 0–1 | 0.394 | 0.313 |
| Justifiable: Abortion | 943 | 0–1 | 0.947 | 0.115 |
| Justifiable: Homosexuality | 948 | 0–1 | 0.974 | 0.096 |
| Justifiable: Sex before marriage | 947 | 0–1 | 0.935 | 0.177 |
| Justifiable: Underage teenage sex | 940 | 0–1 | 0.823 | 0.251 |
| Justifiable: Divorce | 947 | 0–1 | 0.930 | 0.152 |
| Justifiable: Spank children | 950 | 0–1 | 0.971 | 0.116 |
| Justifiable: Beat wife | 948 | 0–1 | 0.991 | 0.083 |
| Justifiable: Prostitution (selling) | 942 | 0–1 | 0.178 | 0.249 |
| University for boys | 950 | 0–1 | 0.988 | 0.089 |
| Jobs for men | 949 | 0–1 | 0.990 | 0.083 |
| Men better political leaders | 949 | 0–1 | 0.989 | 0.083 |
| Migrants: Live here | 985 | 0–1 | 0.850 | 0.188 |
| Migrants: Enrich culture | 985 | 0–1 | 0.794 | 0.196 |
| Migrants: Better country | 983 | 0–1 | 0.738 | 0.224 |
| Religiosity | 994 | 0–1 | 0.379 | 0.206 |
| Religious affiliation |  |  |  |  |
| None | 989 | 0/1 | 0.705 |  |
| Church of Sweden | 989 | 0/1 | 0.226 |  |
| Other Christian | 989 | 0/1 | 0.057 |  |
| Other affiliations | 989 | 0/1 | 0.012 |  |

Note: Categorical variables coded 0/1.

**TABLE S2.** Descriptive statistics for demographic variables from external datasets. MigraMed Healthcare Providers Study, Sweden, 2021.

|  | **ESS2002** | | | **EVS2017** | | | **ISSP20018** | | | **WVS2011** | | |
| --- | --- | --- | --- | --- | --- | --- | --- | --- | --- | --- | --- | --- |
|  | **N** | **Mean** | **Sd** | **N** | **Mean** | **Sd** | **N** | **Mean** | **Sd** | **N** | **Mean** | **Sd** |
| Gender |  |  |  |  |  |  |  |  |  |  |  |  |
| Male | 1907 | 0.492 |  | 1192 | 0.507 |  | 1777 | 0.472 |  | 1206 | 0.500 |  |
| Female | 1907 | 0.508 |  | 1192 | 0.493 |  | 1777 | 0.528 |  | 1206 | 0.500 |  |
| Education level |  |  |  |  |  |  |  |  |  |  |  |  |
| Below bachelor | 1906 | 0.831 |  | 1180 | 0.705 |  | 1756 | 0.675 |  | 1181 | 0.811 |  |
| Bachelor and above | 1906 | 0.169 |  | 1180 | 0.295 |  | 1756 | 0.325 |  | 1181 | 0.189 |  |
| Age | 1907 | 47.273 | 17.316 | 1186 | 45.354 | 17.346 | 1777 | 53.730 | 16.293 | 1206 | 47.624 | 18.298 |

**TABLE S3.** Descriptive statistics for value questions from external datasets. MigraMed Healthcare Providers Study, Sweden, 2021.

| **Variable (study)** | **Dataset** | **Variable (dataset)** | **Sweden** | | | **Sub-sample^a^** | | | **p-values^b^** |
| --- | --- | --- | --- | --- | --- | --- | --- | --- | --- |
|  |  |  | **N** | **Mean** | **Sd** | **N** | **Mean** | **Sd** |  |
| Justifiable: Abortion | EVS2017 | F120 | 1167 | 0.803 | 0.283 | 194 | 0.891 | 0.191 | 0.000 |
| Justifiable: Homosexuality | EVS2017 | F118 | 1164 | 0.854 | 0.291 | 196 | 0.972 | 0.125 | 0.000 |
| Justifiable: Sex before marriage | WVS2011 | V206 | 1164 | 0.879 | 0.256 | 188 | 0.958 | 0.140 | 0.000 |
| Justifiable: Divorce | EVS2017 | F121 | 1183 | 0.843 | 0.254 | 197 | 0.920 | 0.164 | 0.000 |
| Justifiable: Spank children | WVS2011 | V209 | 1183 | 0.940 | 0.181 | 188 | 0.978 | 0.126 | 0.006 |
| Justifiable: Beat wife | WVS2011 | V208 | 1182 | 0.956 | 0.167 | 188 | 0.981 | 0.125 | 0.053 |
| University for boys | EVS2017 | D060 | 1188 | 0.932 | 0.150 | 197 | 0.984 | 0.081 | 0.000 |
| Jobs for men | EVS2017 | C001_01 | 1189 | 0.898 | 0.183 | 197 | 0.958 | 0.134 | 0.000 |
| Men better political leaders | EVS2017 | D059 | 1181 | 0.887 | 0.203 | 196 | 0.968 | 0.119 | 0.000 |
| Migrants: Live here | ESS2002 | imdfetn | 1824 | 0.699 | 0.234 | 160 | 0.806 | 0.198 | 0.000 |
| Migrants: Culture undermined/enriched | ESS2002 | imueclt | 1843 | 0.688 | 0.232 | 168 | 0.814 | 0.157 | 0.000 |
| Migrants: Sweden worse/better country | ESS2002 | imwbcnt | 1842 | 0.587 | 0.237 | 166 | 0.697 | 0.206 | 0.000 |
| Religiosity | ISSP2018 | v48 | 1711 | 0.359 | 0.231 | 289 | 0.368 | 0.218 | 0.529 |
| Religious affiliation |  |  |  |  |  |  |  |  |  |
| None | ISSP2018 | SE_RELIG | 1764 | 0.300 |  | 296 | 0.318 |  | 0.553 |
| Church of Sweden | ISSP2018 | SE_RELIG | 1764 | 0.641 |  | 296 | 0.622 |  | 0.530 |
| Other Christian | ISSP2018 | SE_RELIG | 1764 | 0.045 |  | 296 | 0.041 |  | 0.711 |
| Other affiliations | ISSP2018 | SE_RELIG | 1764 | 0.014 |  | 296 | 0.020 |  | 0.376 |

a. High-educated (bachelor and above) women no older than 67.

b. p-values from a two-sided t-test between Sweden and Sub-sample.

**TABLE S4.** Average marginal effects from multinomial logistic regression of religious affiliation. MigraMed Healthcare Providers Study, Sweden, 2021.

|  | **None** | **Church of Sweden** | **Other Christian** | **Other affiliations** |
| --- | --- | --- | --- | --- |
| Clinical years | -0.009 | 0.011* | -0.002 | 0.000 |
|  | (0.005) | (0.004) | (0.002) | (0.001) |
| (Clinicalyears^2)*100 | 0.024* | -0.024* | 0.003 | -0.003 |
|  | (0.012) | (0.011) | (0.006) | (0.004) |

Note: Robust standard errors in parentheses. N=974. Pseudo-r^2^ =0.0065.

*p<0.05

**TABLE S5.** Justify: Abortion among all healthcare providers in EVS2017. MigraMed Healthcare Providers Study, Sweden, 2021.

|  | **N** | **Mean** | **Sd** |
| --- | --- | --- | --- |
| Justify: Abortion | 166 | 0.846 | 0.254 |

Note: Definition of healthcare providers based on ISCO08 2-digit codes 22, 32, and 53.

**TABLE S6.** Religion among all healthcare providers in ISSP2018. MigraMed Healthcare Providers Study, Sweden, 2021.

|  | **N** | **Mean** | **Sd** |
| --- | --- | --- | --- |
| Religiosity | 222 | 0.372 | 0.244 |
| Religious affiliation |  |  |  |
| None | 229 | 0.314 |  |
| Church of Sweden | 229 | 0.594 |  |
| Other Christian | 229 | 0.079 |  |
| Other affiliations | 229 | 0.013 |  |

Note: Definition of healthcare providers based on ISCO08 4-digit codes 1342, 1343, 2211, 2212, 2221, 2222, 2261, 2262, 2263, 2264, 2269, 3211, 3212, 3213, 3214 3251, 3254, 3255, 3257, 3258, 5320, 5321, 5322, and 5329.
